# Atrial myxoma embolization of the basilar artery presenting with a convulsive seizure: Case report

**DOI:** 10.1097/MD.0000000000036138

**Published:** 2023-11-24

**Authors:** Yaning Xu, Jiaojin Jiang, Meng Zhang

**Affiliations:** a Department of Neurology, Army Medical Center of PLA, Army Military Medical University, Chongqing, China.

**Keywords:** atrial myxoma, basilar artery, case report, endovascular treatment, seizure

## Abstract

**Background:**

Basilar artery occlusion (BAO) is a rare cause of convulsive seizure. Such patients who are treated for epilepsy will miss the optimal time for treatment. Atrial myxoma is a rare cause of stroke and should be surgically removed as soon as possible after diagnosis.

**Case summary:**

We report a patient who presented with convulsions as the initial symptom and was diagnosed with BAO by computed tomographic angiography. After transthoracic echocardiogram, the cause of the disease was diagnosed as atrial myxoma. The patient recovered well after endovascular treatment and resection of the atrial myxoma.

**Conclusion:**

A small number of patients with BAO present with convulsive seizures. It is very important to make a timely diagnosis. Direct thrombaspiration may be the best choice for basilar artery cardioembolization, and thrombectomy for distal moderate vascular occlusion in posterior circulation is feasible. Atrial myxoma is a rare cause of cardioembolic stroke and should be resected as soon as possible to prevent further embolic complications.

## 1. Introduction

Basilar artery occlusion (BAO) is a fatal ischemic stroke that accounts for about 1%^[1]^ of all strokes and 10% of all large vessel occlusions that is amendable to endovascular treatment (EVT).^[2]^ Clinical evidence on thrombectomy for BAO has gradually increased,^[3–5]^ but clinical studies of posterior circulation distal moderate vessel thrombectomy are still lacking. Convulsive seizure is a rare initial presentation in BAO patients. The most frequent causes of BAO are atherosclerotic occlusions and embolic occlusions from cardiac. Atrial myxoma is a rare cause of cardiac embolism. The cerebrovascular is the most location for embolization from a myxoma, and the associated stroke prevalence is 21% to 29%.^[6]^

## 2. Case presentation

### 2.1. Patient description

A previously healthy 50-year-old man presented to the emergency department 7 hours after his wife noted that he experienced sudden unconsciousness and convulsions of the extremities. The symptoms occurred 3 times, each lasting about 4 to 7 minutes. When the patient arrived at the hospital, his symptoms had been partly relieved. The patient had been smoking approximately 20 cigarettes a day for more than 30 years. He had no history of hypertension, diabetes, atrial fibrillation or other internal diseases, and the patient did not have a history of food or drug allergies.

**Figure 1.**
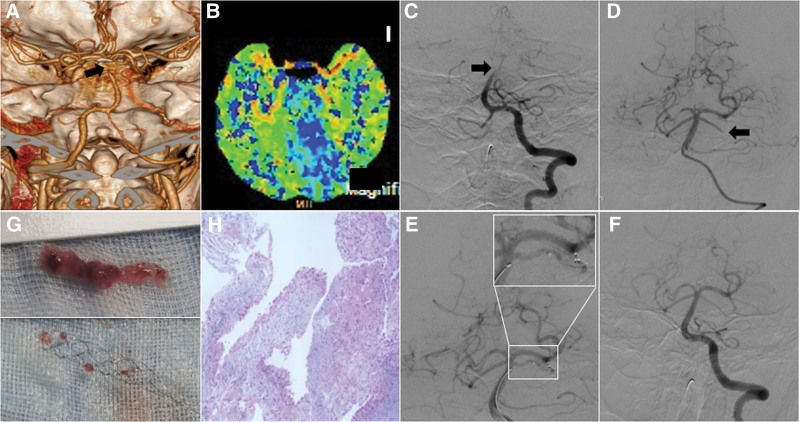
Perioperative imaging of endovascular treatment. (A) Computed tomographic angiography shows BA occlusion (arrow). (B) Computed tomographic perfusion shows hypoperfusion in the left cerebellum and occipital lobe. (C) DSA reconfirm the BA occlusion (arrow). (D) LSCA remained occluded after BA thrombaspiration (arrow). (E) Stent retriever thrombectomy in LSCA. (F) DSA after thrombectomy shows recanalization of the BA and LSCA. (G and H) Gross appearance and histology of the thrombus. BA = basilar artery, DSA = digital subtraction angiograph, LSCA = left superior cerebellar artery.

General physical examination was within normal limits, and initial blood pressure was 122/57 mm Hg. Upon neurologic examination, he was awake, and able to follow all commands, but displayed partial motor aphasia. Cranial nerves testing was normal. On motor examination, the strength of the extremities was normal, but the left upper and lower extremities showed ataxia. Bilateral sensory examination was symmetrical. The patient was given an National Institutes of Health Stroke Scale score of 4. Initial computed tomographic angiography showed an occlusion in the top of the basilar artery, and CT perfusion showed hypoperfusion in the left cerebellum and occipital lobe (Fig. 1A and B).

### 2.2. Endovascular treatment

EVT was performed considering the possibility of symptom exacerbation. During the procedure, digital subtraction angiography reconfirmed the basilar artery was occluded (Fig. 1C). An ACE 068 catheter was used for aspiration to remove the thrombus. A thrombus about 1.5 cm in length was removed and digital subtraction angiography showed the left superior cerebellar artery (LSCA) was still occluded (Fig. 1D). The neurointerventionist delivered a Solitaire 4 × 20 mm stent into the LSCA for a second thrombectomy (Fig. 1E). Several microthrombi were removed and achieved complete successful reperfusion (Fig. 1F and G). Pathologic examination of these thrombus suspected to atrial myxoma (Fig. 1H).

### 2.3. Diagnosis and treatment of etiology

**Figure 2.**
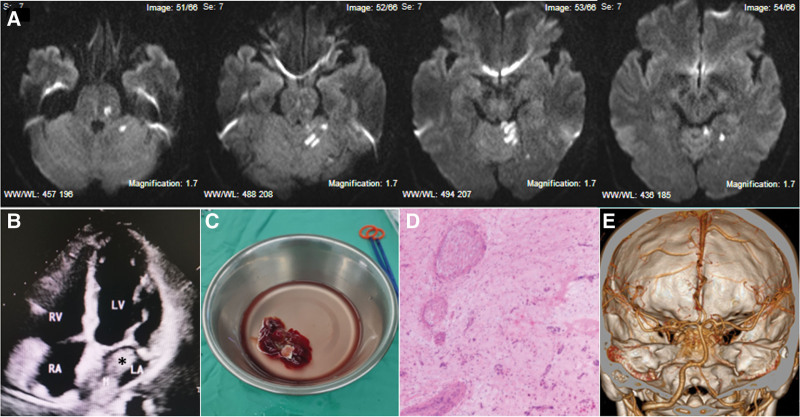
Atrial myxoma before and after cardiac surgery and 3 months follow-up imaging. (A) Diffusion weighted magnetic resonance imaging shows ischemic infarctions in the left pons, cerebellum, and occipital lobe. (B) Transthoracic echocardiogram illustrates atrial myxoma in the left atrium (asterisk). (C and D) Gross appearance and histology of the resected myxoma tissue. (E) Computed tomographic angiography at 3 months after discharge shows basilar artery was normal.

Postintervention magnetic resonance imaging showed new ischemic infarctions in the left pons, cerebellum, and occipital lobe (Fig. 2A). To determine the cause of stroke, a transthoracic echocardiogram was done, which showed a large (4.4 × 2.4 cm) mass in the left atrium (Fig. 2B). This finding further increases the likelihood of atrial myxoma, which was thought to be the cause of BAO. To prevent recurrent embolic events, the patient was transferred to the department of cardiac surgery the day after EVT for myxoma resection. A fragile, translucent, brown tissue with a size of 5.8 × 3.4 × 3.2 cm was removed (Fig. 2C). Histopathological examination reconfirmed the atrial myxoma (Fig. 2D).

### 2.4. Outcome and follow-up

After surgery, the patient continued to be hospitalized for 1 week. During this period, he did not experience any further convulsive seizures or other neurological symptoms. At 3 months of follow-up, the patient recovered well with an National Institutes of Health Stroke Scale score of 0 and computed tomographic angiography showed the basilar artery was normal (Fig. 2E). The patient and his family members were satisfied with the treatment and their daily life was barely affected after discharge.

## 3. Discussion

The clinical presentation of BAO varies widely, ranging from mild transient ischemic attacks to devastating strokes, but convulsant onset is rare. The possible mechanisms of a convulsive seizure include disruption of inhibitory projections from cortex to brainstem, acute ischemic injury of the corticospinal, vestibulospinal, and reticulospinal tracts, and destruction of the medial temporal lobe and occipital lobe functional areas supplied by posterior cerebral artery.^[7]^ BAO in convulsant onset is difficult to diagnose, and this clinical symptom is often diagnosed as epilepsy. Antiepileptic therapy results in delayed treatment of BAO. Timely identification of BAO by imaging examination is very important. Current clinical studies have shown that EVT can improve the outcome of patients with BAO.^[3–5]^ Direct thrombaspiration may be a more effective approach, which can improve the rate of complete reperfusion, reduces procedure duration, and reduce the risk of distal embolization of fragmented thrombi.^[8]^ In this patient, the main part of the myxoma was aspirated during the first thrombectomy, which indicates that thrombaspiration is also efficient for thrombectomy of fragile thrombi.

Because the myxoma is fragile, the thrombus was not completely removed at one time, and the LSCA still occluded. For distal moderate vascular occlusion, EVT is not the first-line recommendation, and few cases of SCA thrombectomy have been reported. The vertebrobasilar artery is generally more tortuous than the carotid artery, and the SCA is at right angles or even acute angles to the basilar artery. These factors can increase the risk of interventional complications such as dissection, perforation, vasospasm, and bleeding caused by traction.^[9]^ However, SCA occlusion will lead to cerebellar infarction. When the infarct size is large, cerebellar swelling is obvious, which may compress the brainstem and fourth ventricle, leading to disturbance of consciousness, acute hydrocephalus, and even brain hernia. About one-third of patients are left with severe disability and need long-term care.^[10]^ The LSCA thrombectomy was performed by our neurointerventionist using the Solitaire AB With Intracranial Support Catheter for Mechanical Thrombectomy technology with no complications.

Atrial myxoma is the most common tumor of the heart and a rare cause of cardioembolic stroke. The tumor size, location, mobility, and irregular surface are related to embolic events. Early resection of the tumor after diagnosis is critical, but cardiac surgery requires systemic anticoagulation and cardiopulmonary bypass. During the procedure, patients with acute ischemic stroke are at increased risk of intracranial hemorrhage. Therefore, some investigators have proposed bridging therapy with antiplatelet or anticoagulant therapy to delay the surgery.^[11]^ It is important to recognize that antiplatelet or anticoagulant therapy is not a substitute for atrial myxoma resection, and the risk of another embolic event increases over time.^[12]^

Fortunately, the patient was diagnosed timely after admission. Because the cerebral infarction volume was small and there were no contraindications for cardiac surgery, the patient underwent atrial myxoma resection on the second day after EVT, effectively preventing the recurrence of embolic complications.

## 4. Conclusion

A small number of patients with BAO present with convulsive seizure. It is very important to make a timely diagnosis. Direct thrombaspiration may be the best choice for basilar artery cardioembolization, and thrombectomy for distal moderate vascular occlusion in posterior circulation is feasible. Atrial myxoma is a rare cause of cardioembolic stroke and should be resected as soon as possible to prevent further embolic complications.

## Author contributions

**Investigation:** Yaning Xu, Jiaojin Jiang.

**Visualization:** Yaning Xu.

**Writing – original draft:** Yaning Xu.

**Writing – review & editing:** Yaning Xu, Jiaojin Jiang, Meng Zhang.
